# Effectiveness of Gastrocnemius-Soleus Stretching Program as a Therapeutic Treatment of Plantar Fasciitis

**DOI:** 10.7759/cureus.22532

**Published:** 2022-02-23

**Authors:** Muhammad Asad Arif, Sohail Hafeez

**Affiliations:** 1 Orthopedics and Trauma, Shifa International Hospital Islamabad, Islamabad, PAK

**Keywords:** heel spur syndrome, jogger's heel, plantar fasciopathy, plantar fasciosis, pain, chronic plantar fasciitis, tendoachilles stretching exercises, gastrocnemius-soleus stretching program

## Abstract

Introduction: Plantar fasciitis is a painful ailment that causes frustration to both the patient and physician. Stretching exercises targeting the plantar fascia are an excellent therapy option for plantar fasciitis.

Objective: To compare the outcome of a gastrocnemius-soleus stretching program versus tendo Achilles stretching exercises for the management of chronic plantar fasciitis.

Methods: Patients aged 30-70 years of either gender with chronic plantar fasciitis were included and randomly divided into two groups. In group A, the gastrocnemius-soleus stretching program was applied, whereas in group B, the tendo Achilles stretching exercises were adopted. The pain score was determined at baseline and after eight weeks, and the change in pain score was calculated. All information was noted in the proforma and then entered and analyzed in the Statistical Package for the Social Sciences (SPSS) software version 21 (International Business Machines (IBM), New York, United States). An independent-samples *t*-test was conducted to compare the mean change in pain score in both groups. A P value of <0.05 was considered significant.

Results: The mean age of the patients in the gastrocnemius-soleus stretching group was 48.70 ± 9.80 years, whereas that in the tendo Achilles stretching exercises group was 48.63 ± 8.43 years. Group A included 16 (53.3%) men and 14 (46.7%) women, whereas in group B, there were 15 (50%) men and 15 (50%) women. The mean change in pain score in group A was 2.57 ± 1.01, whereas that in group B was 1.77 ± 0.57. The difference in both groups was significant (P < 0.05).

Conclusion: Gastrocnemius-soleus stretching exercises are more effective for reducing the symptoms of plantar fasciitis in the adult population.

## Introduction

The plantar fascia is a thick, multilayered fibrous connective tissue on the sole of the foot that facilitates the formation of the foot’s longitudinal arch [1]. When it comes to moving pressure to the forefoot, a heel lift might be useful [2]. Plantar fasciitis is characterized by inflammation of the plantar fascia’s origin and adjacent perifascial tissues. Despite substantial research into this issue, foot surgeons continue to disagree over the cause and genesis of plantar heel pain, as well as the best treatment option [3].

The connection between the fascia and skeletal muscles to form a bodywide network of multidirectional myofascial continuity calls into question the traditional view of muscles as autonomous actuators [4]. In primary care, conservative therapy is recommended, and many conservative approaches have been discussed in the literature, e.g., shoe inserts, nonsteroidal anti-inflammatory drugs (NSAIDs), night splints, stretching exercises, extracorporeal shockwave therapy (ESWT), corticosteroid injection, botulinum toxin (botox) injection, taping, and casting [5]. As part of the initial therapy for plantar fasciitis, the gastrocnemius-soleus muscles are frequently stretched to increase the dorsiflexion range of motion [6].

The inflexibility of the gastrocnemius-soleus complex has been linked to excessive pronation and overcompensation of the plantar fascia at the first metatarsal phalangeal joint, which increases tension at the medial calcaneal insertion [6]. Thus, gastrocnemius-soleus stretches are thought to be advantageous in the early stages of a plantar fasciitis therapy or rehabilitation program [6]. This study aimed to compare the outcome of a gastrocnemius-soleus stretching program versus tendo Achilles stretching exercises for the management of chronic plantar fasciitis. Previous studies have demonstrated that a gastrocnemius-soleus stretching program is more effective in reducing pain as compared with tendo Achilles stretching exercises [6]. However, there have been no local studies on this topic confirming the beneficial role of gastrocnemius-soleus stretching. Thus, we conducted the current study to compare the outcome of a gastrocnemius-soleus stretching program versus that of tendo Achilles stretching exercises for the management of chronic plantar fasciitis.

## Materials and methods

Sample and study characteristics

This randomized controlled trial was conducted in the Department of Orthopedic Surgery, Shifa International Hospital, Islamabad, over a period of approximately six months (i.e., from September 19, 2020, to March 19, 2021). We estimated that 60 subjects were required for the study (i.e., 30 cases in each group) to maintain a 95% confidence level and 80% study power and to detect a mean change in pain score of 2.50 ± 0.861 in the gastrocnemius-soleus stretching program and 1.71 ± 1.006 with tendo Achilles stretching exercises for the management of chronic plantar fasciitis.

Patients were recruited by applying a nonprobability, consecutive sampling technique. The inclusion criteria were patients aged 30-70 years of either gender who were experiencing chronic plantar fasciitis, defined as presenting with a complaint of severe pain for more than three months upon placement of the foot on a hard surface or while walking as well as the presence of maximum pain upon palpation of the origin of the plantar fascia on the medial calcaneal tubercle on clinical examination. Patients with anatomical abnormalities of the foot or ankle (changes in deep tendon reflexes, motor, or sensory deficit) or a history of systemic inflammatory disease, diabetes, heel surgery, or heel pain not consistent with the proximal plantar fasciitis were excluded from the study.

Study methods

After obtaining approval from the ethical board of the hospital, 60 patients were recruited from outpatient department and provided informed consent. Shifa International Hospital Ltd. (SIH), Shifa Tameer-e-Millat University (STMU) issued approval 338-1158-2020. The demographic profiles of the patients were also obtained. The patients were randomly divided into two equal groups by applying the lottery method. In group A, the gastrocnemius-soleus stretching program was applied, whereas in group B, the patients were advised to apply the tendo Achilles stretching exercises. The pain score at baseline was determined using the Visual Analog Scale. The patients were followed up in the out-patient department (OPD) for eight weeks. After eight weeks, their pain score was reevaluated using the Visual Analog Scale and the change in pain score from baseline was calculated. All data were gathered in proforma.

Data analysis

The data were entered and analyzed using the SPSS software version 21 (International Business Machines (IBM), New York, United States). Numerical variables such as age, body mass index (BMI), duration of symptoms, and pre- and posttreatment pain scores were expressed as mean ± standard deviation. Categorical variables such as gender and lateral side were expressed as frequency and percentage. An independent-samples t-test was employed to compare the mean change in pain score in both groups. A P value ≤ 0.05 was considered significant. The data were stratified for age, gender, body mass index (BMI), and duration of symptoms. After stratification, an independent-samples t-test was applied to compare the mean change in pain scores in both groups in each strata. P ≤ 0.05 was considered significant.

## Results

The mean age of the patients in group A (gastrocnemius-soleus stretching) was 48.70 ± 9.80 years, whereas that in group B (tendo Achilles stretching) was 48.63 ± 8.43 years. There were 16 (53.3%) men and 14 (46.7%) women in group A, whereas in group B, there were 15 (50%) men and 15 (50%) women. In group A, the mean body mass index (BMI) of patients was 26.43 ± 2.21 kg/m^2^, whereas that in group B was 27.30 ± 1.99 kg/m^2^. The mean duration of symptoms was 2.20 ± 0.89 months in group A, whereas it was 1.97 ± 0.81 months in group B. In group A, 11 (36.7%) patients had symptoms in the left foot and 10 (33.3%) had symptoms in the right foot, whereas nine (30%) patients had symptoms in both feet. In group B, nine (30%) patients had symptoms in the left foot and 14 (46.7%) had symptoms in the right foot, whereas seven (23.3%) patients had symptoms in both feet (Table 1).

**Table 1:** Comparison of change in pain score in both groups during follow-up

	Group		P Value
	Gastrocnemius-Soleus Stretching	Tendo Achilles Stretching Exercises	
n	30	30	
Pain at baseline	4.67 ± 0.92	4.30 ± 0.60	0.073
Pain after eight weeks	2.10 ± 1.03	2.53 ± 0.57	0.048
Change in pain score	2.57 ± 1.01	1.77 ± 0.57	0.000
P value (paired-sample t-test)	0.000	0.000	

The mean pain score of patients in group A was 4.67 ± 0.92, which was reduced to 2.10 ± 1.03 after eight weeks; this reduction in the mean pain score was significant in this group (change = 2.57 ± 1.01). In group B, the mean pain score was 4.30 ± 0.60, which was reduced to 2.53 ± 0.57 after eight weeks, representing a significant reduction in the mean pain score (change = 1.77 ± 0.57) in this group. The difference in both groups was significant after four weeks, and there was a significantly greater reduction in the pain score with gastrocnemius-soleus stretching exercises (P < 0.05; Table 2).

**Table 2:** Comparison of change in pain score in both groups during the follow-up for effect modifiers

		Group		P Value
		Gastrocnemius-Soleus Stretching	Tendo Achilles Stretching Exercises	
Age 30-50 years	n	19	17	0.001
	Change in pain score	2.68 ± 0.95	1.76 ± 0.56	
Age 51-70 years	n	11	13	0.112
	Change in pain score	2.36 ± 1.12	1.77 ± 0.60	
Male gender	n	16	15	0.001
	Change in pain score	2.94 ± 1.06	1.80 ± 0.56	
Female gender	n	14	15	0.119
	Change in pain score	2.14 ± 0.77	1.73 ± 0.59	
Normal body mass index (BMI)	n	8	3	0.241
	Change in pain score	2.25 ± 0.46	2.67 ± 0.58	
Overweight	n	20	23	0.001
	Change in pain score	2.70 ± 1.17	1.70 ± 0.47	
Obese	n	2	4	0.132
	Change in pain score	2.50 ± 0.71	1.50 ± 0.58	
Duration one to two months	n	19	23	0.007
	Change in pain score	2.53 ± 0.96	1.78 ± 0.60	
Duration three to four months	n	11	7	0.058
	Change in pain score	2.64 ± 1.12	1.71 ± 0.49	

In patients aged 30-50 years, there was a significantly greater mean change with gastrocnemius-soleus stretching exercises as compared with the tendo Achilles stretching exercises (i.e., 2.68 ± 0.95 versus 1.76 ± 0.56, P < 0.05). However, for those aged 51-70 years, the mean change did not significantly differ in both groups (i.e., 2.36 ± 1.12 versus 1.77 ± 0.60, P > 0.05). The mean change in male patients was significantly better with gastrocnemius-soleus stretching exercises as compared with tendo Achilles stretching exercises (i.e., 2.94 ± 1.06 versus 1.80 ± 0.56, P < 0.05). However, the mean change in women did not significantly differ in both groups (i.e., 2.14 ± 0.77 versus 1.73 ± 0.59, P > 0.05). The mean change in patients with normal BMI did not significantly differ in both groups (i.e., 2.25 ± 0.46 versus 2.67 ± 0.58, P > 0.05). However, the mean change in pain score among overweight patients was better with gastrocnemius-soleus stretching exercises as compared with tendo Achilles stretching exercises (i.e., 2.70 ± 1.17 versus 1.70 ± 0.47, P < 0.05). The mean change in obese patients did not significantly differ in both groups (i.e., 2.50 ± 0.71 versus 1.50 ± 0.58, P > 0.05). We also observed that in patients with a symptom duration of one to two months, the mean change in pain score was significantly better with gastrocnemius-soleus stretching exercises than with tendo Achilles stretching exercises (i.e., 2.53 ± 0.96 versus 1.78 ± 0.60, P < 0.05). However, in patients who had a symptom duration of three to four months, the difference was insignificant in both groups (i.e., 2.64 ± 1.12 versus 1.71 ± 0.49, P > 0.05; Table 3).

**Table 3:** Patient demographics BMI: body mass index.

	Group	
	Gastrocnemius-Soleus Stretching	Tendo Achilles Stretching Exercises
n	30	30
Age	48.70 ± 9.80	48.63 ± 8.43
Gender		
Male	16 (53.3%)	15 (50%)
Female	14 (46.7%)	15 (50%)
BMI	26.43 ± 2.21	27.30 ± 1.99
Duration of symptoms	2.20 ± 0.89	1.97 ± 0.81
Lateral side		
Left	11 (36.7%)	9 (30%)
Right	10 (33.3%)	14 (46.7%)
Bilateral	9 (30%)	7 (23.3%)

## Discussion

Plantar fasciitis is a frequent medical ailment that affects the back of the foot [7]. It affects about 10% of the population over the course of their lives [8]. Its hallmark signs and symptoms include discomfort in the first step in the morning for at least 10 months. Plantar fasciitis is also known as heel spur syndrome, painful heel syndrome, runner’s heel, subcalcaneal discomfort, calcaneodynia, and calcaneal periostitis [7,9].

Plantar fasciitis is one of the commonest foot problems and results in around a million doctor visits each year [10]. Proximal plantar fasciitis is the most common cause of heel pain, and it affects more than two million Americans each year [11]. Stretching exercises constitute one of the trademark methods of conservative therapy. These exercises decrease tension on the plantar fascia and tight Achilles tendon. In our study, we used only one broad stretching modality, i.e., gastrocnemius-soleus tendo Achilles stretching, which appears to be effective, inexpensive, and simple to implement in a patient-centered treatment protocol for chronic plantar fasciitis, and the results are promising [12,13].

For the treatment of symptoms of proximal plantar fasciitis, a program of non-weight-bearing stretching exercises specific to the plantar fasciitis is preferable over atypical program of weight-bearing Achilles tendon-stretching activities. These findings offer a nonoperative therapy option for individuals with persistent, debilitating plantar heel pain that differs from the current standard of care [14].

In our trial, we observed that patients who participated in a gastrocnemius-soleus stretching program had a mean pain score of 4.67 ± 0.92, which was reduced to 2.10 ± 1.03 after eight weeks, indicating a significant reduction in the mean pain score (change = 2.57 ± 1.01) in this group. On the other hand, in the group who performed tendo Achilles stretching exercises, the mean pain score was 4.30 ± 0.60, which was significantly reduced to 2.53 ± 0.57 after eight weeks (change = 1.77 ± 0.57). The difference in both groups was significant after four weeks, and there was a significantly greater reduction in pain score with gastrocnemius-soleus stretching exercises (P < 0.05) because of the broader tissue targeting as compared to the tendo Achilles stretching exercises.

Jha et al. conducted a similar trial and found that the mean reduction in pain score (on a 10-cm pain scale) was 2.50 ± 0.861 with gastrocnemius-soleus stretching program, whereas it was 1.71 ± 1.006 with tendo Achilles stretching exercises for the management of chronic plantar fasciitis (P < 0.05) [14]. Thus, the gastrocnemius-soleus stretching program was found to be a more appropriate exercise for the resolution of symptoms of plantar fasciitis. Physical therapies used in various studies have proven to be effective to varying degrees, either for the reduction of pain or alleviation of the symptoms of plantar fasciitis [15].

## Conclusions

Based on the results of this trial, we observed and concluded that gastrocnemius-soleus stretching exercise is more effective than other modalities in reducing symptoms of plantar fasciitis in the adult population. Thus, we recommend the application of gastrocnemius-soleus stretching exercises to resolve plantar fasciitis in adults. However, further trials should be conducted to confirm these results, as this study was conducted on a limited sample size.
